# Foodborne Botulism, Canada, 2006–2021^1^

**DOI:** 10.3201/eid2909.230409

**Published:** 2023-09

**Authors:** Richard A. Harris, Christine Tchao, Natalie Prystajecky, Kelly Weedmark, Yassen Tcholakov, Manon Lefebvre, John W. Austin

**Affiliations:** Health Canada, Ottawa, Ontario, Canada (R.A. Harris, K. Weedmark, J.W. Austin);; British Columbia Centre for Disease Control Public Health Laboratory, Vancouver, British Columbia, Canada (C. Tchao, N. Prystajecky);; McGill University, Montreal, Quebec, Canada (Y. Tcholakov);; Nunavik Regional Board of Health and Social Services, Kuujjuaq, Quebec, Canada (M. Lefebvre)

**Keywords:** botulism, Clostridium botulinum, bacteria, food safety, foodborne illness, foodborne botulism, botulinum toxins, outbreak, serotype, antitoxin, Canada

## Abstract

During 2006–2021, Canada had 55 laboratory-confirmed outbreaks of foodborne botulism, involving 67 cases. The mean annual incidence was 0.01 case/100,000 population. Foodborne botulism in Indigenous communities accounted for 46% of all cases, which is down from 85% of all cases during 1990–2005. Among all cases, 52% were caused by botulinum neurotoxin type E, but types A (24%), B (16%), F (3%), and AB (1%) also occurred; 3% were caused by undetermined serotypes. Four outbreaks resulted from commercial products, including a 2006 international outbreak caused by carrot juice. Hospital data indicated that 78% of patients were transferred to special care units and 70% required mechanical ventilation; 7 deaths were reported. Botulinum neurotoxin type A was associated with much longer hospital stays and more time spent in special care than types B or E. Foodborne botulism often is misdiagnosed. Increased clinician awareness can improve diagnosis, which can aid epidemiologic investigations and patient treatment.

Human foodborne botulism is a neuroparalytic disease that results from ingestion of foods containing botulinum neurotoxin (BoNT) serotypes A, B, E, or F, produced by *Clostridium botulinum* groups I and II or, rarely, neurotoxigenic strains of *C. baratii* type F or *C. butyricum* type E (*1*). BoNTs prevent muscle contraction through cleavage of the proteins responsible for fusion of acetylcholine-containing synaptic vesicles in nerve terminals at neuromuscular junctions (*2*). 

Clinical symptoms of botulism include symmetric cranial nerve palsies of the eyes, mouth, and throat. Paralysis can descend to the diaphragm, causing respiratory arrest that can necessitate use of mechanical ventilation (*1*). In some instances, patients can take months or years to recover from prolonged disability caused by skeletal muscle paralysis (*3*). Treatment options are limited to use of botulinum antitoxin (BAT) that binds to and neutralizes circulating BoNTs (*4*). BAT is especially effective when administered early (*5*), and its use should be based on clinical diagnosis, rather than waiting for results from diagnostic tests.

Manifestations of botulism are classified according to the route of exposure to BoNTs. Wound botulism occurs when *C. botulinum* colonizes an infected wound, and intestinal toxemia botulism occurs in the adult intestinal tract when BoNTs are released in situ (*6*,*7*). Infant botulism is a form of intestinal toxemia botulism that occurs in children <1 year of age (*8*). Foodborne botulism is an acute intoxication resulting from ingestion of BoNTs preformed in foods supporting *C. botulinum* growth*.* *C. botulinum* endospores are widely distributed in soils throughout the world and survive heating processes that inactivate vegetative bacterial cells (*9*). Foods contaminated with viable *C. botulinum* spores can germinate, grow, and produce BoNTs when they are stored under permissive growth conditions, including low oxygen, low acidity (pH >4.6), sufficient temperature (>10°C), and water activity (a_w_ >0.93) (*10*).

Investigations of foodborne botulism provide valuable information regarding food sources and storage conditions that permit *C. botulinum* growth and BoNT production. Previous reports of foodborne botulism in Canada are available, including the periods of 1919–1973 (*11*), 1971–1984 (*12*), and 1985–2005 (*13*). Here, we present a summary of foodborne botulism in Canada during 2006–2021, including incidence over the course of time, geographic distribution by province and territory, BoNT serotype, and food source when available. In addition, we used hospital records that match cases from laboratory-confirmed outbreaks to determine clinical disease outcomes.

## Methods

### Microbiology Laboratory and National Surveillance Data

We examined 2 independent laboratory databases for laboratory-confirmed outbreaks of foodborne botulism during 2006–2021, one maintained by the Botulism Reference Service (BRS) for Canada at Health Canada, Ottawa, Ontario, and the other from British Columbia Centre for Disease Control (BCCDC) Public Health Laboratory (PHL), in Vancouver, British Columbia. BRS receives and tests clinical and food specimens associated with suspected botulism cases from all provinces and territories, when requested. The BCCDC PHL provides clinical and foodborne botulism testing services for British Columbia but also tests specimens from the Yukon. Thus, the 2 databases do not overlap and, when combined, represent all the laboratory‑confirmed outbreaks of botulism in Canada. We extracted information regarding patient age and sex, outbreak date and location, implicated food source, and BoNT serotype from those databases. We also extracted case information from the 2006–2019 Canadian Notifiable Disease Surveillance System (CNDSS) and compared those cases to laboratory data for completeness (*14*). CNDSS maintains basic surveillance on nationally notifiable diseases by collecting voluntarily submitted data from provinces and territories. We calculated the rates of disease per 100,000 population by using population data from Statistics Canada (*15*).

### National Case Definition for Foodborne Botulism

We used the national case definition for confirmed cases of foodborne botulism in Canada to ensure consistency in data recording. That definition is as follows: laboratory confirmation of intoxication with clinical evidence, such as detection of botulinum neurotoxin in serum, stool, gastric aspirate, or food; or isolation of *C. botulinum* from stool or gastric aspirate; or clinical evidence and indication that the client ate the same suspect food as a person with laboratory-confirmed botulism (*16*). Because of the urgency of the disease, 1 case of botulism constitutes an outbreak.

### Laboratory Confirmation of Clinical Cases

Detection of BoNT and isolation of viable *C. botulinum* from food and clinical specimens were performed according to Health Canada standard methods by using a mouse bioassay to detect BoNT in foods and clinical specimens (*17*). BoNT serotype was determined by neutralization of toxicity with serotype-specific antibodies provided by the US Centers for Disease Control and Prevention. If isolates were not obtained from clinical or food specimens, cases caused by *C. baratii* (type F) or *C. butyricum* (type E) might not have been detected.

### Clinical Outcome Data

We retrieved records on patient clinical information by querying the Canadian Institute for Health Information (CIHI) 2005–2021 Discharge Abstract Database (https://www.cihi.ca/en/discharge-abstract-database-metadata-dad) and the 2005–2010 Hospital Morbidity Database (HMDB), which is specific to Quebec (*18*). Data were also collected as part of epidemiologic investigations conducted in Quebec by the Nunavik Regional Board of Health and Social Services (NRBHSS), including records from 2010–2021 that were validated with patient files from the relevant hospitals and were not available in HMDB. We then matched those data to BRS records by age, sex, date of admission, and province of residence. We defined a special care unit in accordance with HMDB as an inpatient unit that is specifically designed, staffed and equipped for the observation and treatment of patients who cannot be cared for in a general acute care unit; these include intensive care units and step-down units (*18*). Formal ethics approval was not required because this study used deidentified healthcare data that were obtained under an agreement with CIHI.

## Results

### Incidence of Foodborne Botulism in Canada

**Figure 1:**
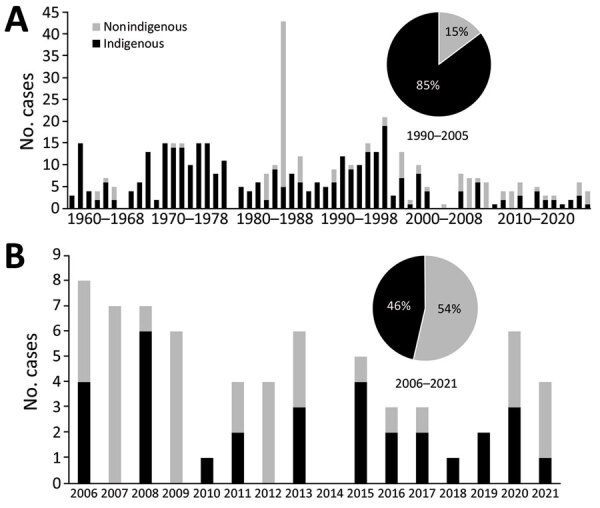
Number of foodborne botulism cases, Canada, 1960–2021. A) Number of cases during 1960–2021; B) detail of number of cases during 2006–2021. Inset pie graphs represent the percentage of cases among non-Indigenous and Indigenous persons.

During 2006–2021, a total of 55 laboratory-confirmed outbreaks of foodborne botulism occurred in Canada, comprising 67 cases (Figure 1). During the reporting period, we determined the average annual incidence of foodborne botulism in Canada was 0.01 case/100,000 population. The CNDSS reported 60 cases of foodborne botulism during 2006–2019, which compares to 55 laboratory-confirmed cases of foodborne botulism from the same reporting period. That discrepancy was not unexpected because the reference laboratories only record laboratory-confirmed cases, but public health authorities include unconfirmed cases or cases that might have been epidemiologically linked but not laboratory tested.

### Geographic Distribution and BoNT Serotype Breakdown

During the reporting period, 31 foodborne botulism cases occurred in Quebec, 15 in Ontario, 6 in Alberta, 5 in Nunavut, 3 in British Columbia, 3 in Northwest Territories, 2 in Manitoba, 1 in Saskatchewan, and 1 in Newfoundland and Labrador (Table 1). Type E was implicated in the most cases (52%, n = 35) across Canada during the reporting period (Table 1). Other serotypes of foodborne botulism across Canada included 16 cases of type A, 11 cases of type B, and 2 cases of type F. Two cases involved clinical samples that were neutralized by multivalent antiserum but were not typed due to insufficient sample. One case was typed as AB because the toxin in the food sample was neutralized by a combination of type A and type B antisera. Indigenous communities represented the most (86%, n = 30) type E cases. Type E was implicated in 21 (68%) cases in Quebec and all (100%) cases in each of Nunavut, the Northwest Territories, and Newfoundland and Labrador.

**Table 1:** Foodborne botulism cases by province or territory and BoNT serotype, Canada, 2006–2021

Province/territory	Serotype, no. cases						Total
	A	B	E	F	AB	Unknown	
Quebec	5	2	21	1	1	1	31
Ontario	4	6	4	1	0	0	15
Alberta	4	1	0	0	0	1	6
Nunavut	0	0	5	0	0	0	5
British Columbia	1	1	1	0	0	0	3
Northwest Territories	0	0	3	0	0	0	3
Manitoba	1	1	0	0	0	0	2
Saskatchewan	1	0	0	0	0	0	1
Newfoundland and Labrador	0	0	1	0	0	0	1
Total	16	11	35	2	1	2	67

### Foods Associated with Outbreaks

Apart from a single outbreak from salmon eggs in British Columbia in 2013, all the outbreaks in Indigenous communities were caused by products from marine mammals, including seal, whale, and walrus, that were incubated in conditions favorable to *C. botulinum* growth and consumed without cooking. Commercial retail foods were responsible for 4 outbreaks, including an international outbreak of contaminated carrot juice in 2006 that affected 2 persons in Canada (*3*) and an outbreak in Canada caused by salted fish in 2012 that affected 3 persons (*19*). The 2 other outbreaks attributed to retail foods were caused by ground beef that affected 2 persons in 2009 and Alfredo sauce that affected 1 person in 2021 (*20*). In those cases, the cooked ground beef was left at room temperature on the stove top, and the Alfredo sauce was recalled because of storage at room temperature by the retailer, despite a label indicating the product should be kept refrigerated (*20*). Of note, no outbreaks from restaurant dining occurred through the reporting period. Home-prepared foods were responsible for only 2 outbreaks, 1 from spaghetti sauce that affected 2 persons in 2006 and 1 from watermelon jelly that affected 1 person in 2011.

### Clinical Outcomes

To examine the health outcomes of foodborne botulism, we cross-referenced cases to 52 (78% matching) hospital records obtained from CIHI and NRBHSS. In 2 instances from the NRBHSS data, the only hospital records available were that the patient died, and in 1 instance the time spent in special care was unknown. The average age of patients was 57.0 years (SD 16.1 years); 27 (52%) were female and 25 (48%) were male. Most case-patients had severe illness: 38 (78%) patients were transferred to special care units, and 35 (70%) required mechanical ventilation. The average length of hospital stay was 48.3 days (SD 84.3 days). The average length of time spent in special care was 36.3 days (SD 72.7 days). Most (52%, n = 27) case-patients were discharged to home without continuing support, but 4 (8%) were discharged to home with support from healthcare workers, 4 (8%) were transferred to continuing care, 9 (17%) were transferred to acute care, and 1 (2%) was transferred to other (palliative) care. In 7 (14%) cases, the patient died.

### Clinical Outcomes by BoNT Serotype

**Figure 2:**
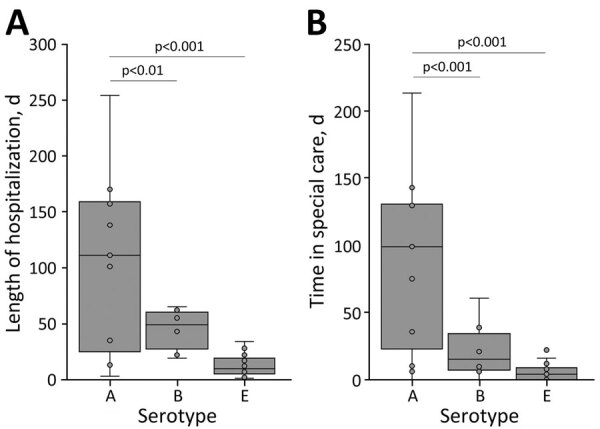
Box and whisker plots of length of hospitalization and special care among persons affected by foodborne botulism serotypes A, B, and E, Canada, 2006–2021. A) Length of hospitalization; B) length of time in a special care unit. The box and whiskers represent the data as quartiles; the whiskers (vertical lines) represent the top and bottom values, the box represents the 1st (bottom) to 3rd (top) quartiles of values, and the horizontal line in the middle of the box represents the median. The circles indicate individual data points including outliers. A single outlier for time in special care occurred for serotype E.

To examine the relationship between BoNT serotype and clinical severity of disease, we performed 1-way analysis of variance tests to compare the serotype of intoxication with the length of hospital stay and time spent in special care (Figure 2). Serotype had a significant effect on the length of hospital stay (p<0.0001) and the length of time spent in special care (p<0.0001). A Tukey honest significant difference post hoc comparison test indicated that cases of type A were associated with significantly longer hospital stays than were type B (p<0.01) or type E (p<0.0001), and type A case-patients spent significantly longer times in special care than did patients with type B (p<0.001) or type E (p<0.0001). We noted no significant difference between types B and E for length of hospital stay (p = 0.17) or time in special care (p = 0.48). We removed 1 case of type A from analysis because the patient was hospitalized for 497 days and that case was identified as an outlier by a 2-sided Grubb test (p<0.01). Type F was not included in this analysis because only a single case that matched hospital records was identified within the reported period. Our results suggest that BoNT type A is associated with more severe clinical outcomes than types B and E. Of note, the 7 deaths during the reporting period were associated with 2 cases of type A, 1 case of each type B and type E, and 1 case of undetermined serotype.

## Discussion

The average annual incidence of foodborne botulism cases in Canada (0.01 case/100,000 population) during 2006–2021 is the same as that of the United States during 2001–2017 (*21*). Canada’s incidence also was less than the overall incidence (0.02 case/100,000 population) in European Union or European Economic Area countries in 2014 and less than incidences in France (0.02–0.03 case/100,000 population) during 2013–2016, Italy (0.03 case/100,000 population) during 1986–2015, Poland (0.04 case/100,000 population) during 2010–2018, and the Republic of Georgia (0.3–0.9 case/100,000 population) during 1980–2002 (*22*–*26*). The average annual incidence of foodborne botulism in Canada has decreased in recent years. During 1985–2005, the incidence was 0.03 case/100,000 population, and during 1971–1984 the incidence was 0.04 case/100,000 population (*12*,*13*).

The reduction in foodborne botulism was most pronounced in Indigenous communities. During 2006–2021, foodborne botulism in Indigenous communities accounted for 46% of all cases, which is a reduction from 85% of all cases for the previous 16-year period of 1990–2005 (Figure 1). In addition, during 2006–2021 the average annual rate of foodborne botulism in Indigenous communities was 1.9 cases/year, but incidence was 6.7 cases/year during 1985–2005 and 8.7 cases/year during 1971–1984 (*12*,*13*). The incidence of type E botulism in Indigenous communities corresponds to the geographic distribution of *C. botulinum* type E spores in shoreline soils along the Hudson Strait and Ungava Bay in northern Quebec (*27*). Contamination occurs during butchering of marine mammal meat, but *C. botulinum* spore germination and production of BoNTs occurs during storage of the traditional Indigenous foods (*27*). Type E strains belong to group II *C. botulinum*, which possesses a lower minimum growth temperature of 2.5°C–3°C than group I strains (*28*), permitting growth in northern climates.

Outbreaks in the Nunavik region of northern Quebec were most associated with igunaq (meat and blubber) and misiraq (a product consisting of oil from the blubber of marine mammals) and occurred most often in the summer months; 70% of cases occurred from July through September. According to the *Qanuilirpitaa*? (How are we now?) 2017 Health Survey, the consumption of country foods (traditional foods that are largely only available in Canada’s far north) did not decline during 2004–2017 and accounted for ≈40% of all meat and fish consumed (*29*). That finding underlines the importance of working closely with Indigenous communities to communicate the risk for disease and the ways of reducing risk, while continuing to practice traditional subsistence activities that are linked with numerous health benefits (*30*,*31*). The NRBHSS collaborates with the Nunavik Hunting, Fishing and Trapping Organization to inform the population about safe traditional food preparation techniques, and symptoms of foodborne botulism intoxications. The NRBHSS recommends chilling butchered meat to below 4°C as soon as possible and storing meat in a freezer (home or community) and to wait to begin the traditional outdoor aging process in the fall when temperatures are cooler. Those interventions might have contributed to the observed decrease of botulism cases in Indigenous communities in Quebec in recent years. In addition, the NRBHSS maintains clinical guidance documents and provides training to clinicians in the region to ensure prompt recognition and management of cases of botulism intoxication.

Foodborne botulism occurs via ingestion of preformed BoNTs in foods contaminated by *C. botulinum*, but identification of a toxic food source remains a significant challenge (*32*). Only 36 (54%) cases were associated with laboratory-confirmed foods in which BoNTs were detected (Table 2). That rate is comparable to the United States, which identified a laboratory-confirmed food vehicle in 47% of all cases during 2001–2017 (*21*), and Italy, which identified a food vehicle in 31% of all laboratory-confirmed cases during 1986–2015 (*24*). The low success rate for food origin tracing might be because most (97%, n = 29) outbreaks without an identified food source involved only a single (sporadic) case. Of those sporadic cases, 24 (83%) had no food submitted for testing. Outbreaks involving several linked individual cases enable epidemiologic identification of foods patients have in common. Of the 8 outbreaks involving >1 case during the reporting period, 7 were traced to a food source. The 1 multicase outbreak that was not traced to a food source was because no food was submitted for testing.

**Table 2:** Foodborne botulism outbreaks, cases, deaths, and serotype, by year and food source, Canada, 2006–2021

Food source	Years	Outbreaks	Cases	Deaths	Serotype
Commercial retail foods					
Carrot juice	2006	1	2	0	A
Ground beef	2009	1	2	0	B
Salted fish	2012	1	3	0	E
Alfredo sauce	2021	1	1	0	AB
Home-prepared foods					
Spaghetti sauce	2006	1	2	0	A
Watermelon jelly	2011	1	1	0	B
Traditionally prepared Indigenous foods (traditional name)					
Blubber in oil (misiraq)	2006–2021	7	8	1	E
Meat and fat (igunaq)	2006–2021	5	10	1	E
Beluga skin (muktuk)	2006–2021	3	3	0	E
Aged meat	2006–2021	3	3	0	A, E
Salmon eggs	2013	1	1	1	E
Unknown*	2006–2021	30	31	4	A, B, E, F

*Food was either not submitted for analysis or was submitted but not found to be toxic.

The data obtained from CIHI and NRBHSS hospital records are consistent with previous reviews indicating that foodborne botulism is a rare disease in the population but is associated with severe clinical outcomes. Recent reports from the World Health Organization (2007–2015), Taiwan (2012–2015), and Greece (1996–2006) have estimated that botulism has one of the lowest overall disability-adjusted life years (accounting for prevalence in the population) of all foodborne illnesses, yet severe botulism ranks as one of the highest disability weights based strictly on clinical outcomes (*33*–*35*). 

In Canada, ≈4 million episodes of domestically acquired foodborne illness occur each year, attributed to 30 known and unknown pathogens (*36*). *C. botulinum* ranks at 28 out of 30 for prevalence (i.e., estimated cases per 100,000 population) but has the highest proportion of hospitalizations and deaths per case of all known pathogens. We found illness caused by BoNT type A was associated with significantly longer hospital stays and more time spent in special care than illness caused by types B and E. That finding is consistent with previous reports showing that BoNT type A has higher rates of severe illness than types B, E, or F, based on a higher proportion of patients requiring mechanical ventilation and longer average hospital stays (*37*,*38*). Another study in the United States (1975–2009) found that type F had a higher mortality rate than types A or B (*39*), although the authors noted that heptavalent antitoxin, which is effective for type F, only became available in 2010.

Two limitations of this study highlight potential opportunities for improved prevention and surveillance of foodborne botulism in Canada: identifying toxic foods associated with outbreaks and comprehensively cross-referencing cases with hospital records. First, the difficulty in identifying a food source can be caused in part by misdiagnosis of botulism as stroke, Guillain-Barré syndrome, or myasthenia gravis. Food history and collection can be delayed by misdiagnosis after an outbreak, resulting in discarding of toxic foods. Improved communication between hospital staff, diagnostic laboratories, and public health officials would help ensure that a food history and sampling is performed for each laboratory-confirmed case of foodborne botulism. The second limitation of this study is the proportion of foodborne botulism cases that were cross-referenced to hospital records. Missing hospital records might in part be a result of the narrow range of years that are available from CIHI databases (2005–2010 for HMDB), which is specific to Quebec and required collaboration with local public health units for records after 2010. In addition, 9 laboratory-confirmed cases did not match any records in CIHI databases, even within the years available. The missing CIHI records for laboratory-confirmed cases of foodborne botulism likely were a result of a missing diagnostic code in the databases. Treatment with BAT was not recorded in CIHI databases as a treatment under the Canadian Classification of Health Interventions (code 8.BB.70.HA-BX); therefore, we found no records for this life-saving therapeutic (*4*,*5*). 

In conclusion, we found that foodborne botulism rates in Canada decreased during 2006–2021 compared with previous years, especially among Indigenous populations. However, cases might have been underreported because of misdiagnosis or lack of appropriate diagnostic coding. Expanding the years available for the HMDB database in CIHI and ensuring the use of proper coding for suspected diagnoses and treatments would help to capture more instances of foodborne botulism in Canada and aid in evaluation of BAT as a therapeutic for patients of this severe illness.
